# Mitochondrial dysfunction as a metabolic checkpoint of immunotherapy response in colorectal cancer

**DOI:** 10.3389/fimmu.2026.1908222

**Published:** 2026-09-21

**Authors:** Lorenzo Tomassini, Marco Colella, Giovanni Monteleone, Carmine Stolfi, Federica Laudisi

**Affiliations:** 1 Department of Systems Medicine, University of Rome “Tor Vergata”, Rome, Italy; 2 Gastroenterology Unit, Policlinico Universitario Tor Vergata, Rome, Italy

**Keywords:** cancer immunotherapy, colorectal cancer, immune checkpoint inhibitors, immune evasion, metabolic reprogramming, mitochondrial dysfunction, T-cell exhaustion, tumor microenvironment

## Abstract

Immunotherapy has markedly improved outcomes across multiple cancer types; however, colorectal cancer (CRC) remains largely refractory, with meaningful responses mostly restricted to tumors harboring deficient mismatch repair and high microsatellite instability (dMMR/MSI-H), which represent only a small fraction of CRC cases. Emerging evidence suggests that mitochondrial dysfunction may be viewed as a metabolic checkpoint, representing a conceptual framework through which mitochondrial-dependent processes regulate antitumor immunity and immunotherapy response in CRC. In tumor cells, mitochondrial reprogramming enhances reliance on oxidative phosphorylation, promotes hypoxia, and drives metabolite accumulation, collectively shaping an immunosuppressive tumor microenvironment. In parallel, tumor-infiltrating T-cells exhibit mitochondrial dysfunction characterized by impaired biogenesis, reduced respiratory capacity and features of exhaustion, thereby limiting the efficacy of immune checkpoint inhibitors (ICIs). In this review, we summarize current insights into the role of mitochondrial pathways in regulating tumor immune escape and T-cell dysfunction in CRC. We further discuss emerging therapeutic strategies aimed at targeting metabolic vulnerabilities to overcome resistance and potentially enhance responses to immunotherapy.

## 1 Introduction

The employment of immune checkpoint inhibitors (ICIs) has transformed the therapeutic approach to treat several immune-inflamed and highly mutated tumors, such as melanoma, non-small cell lung cancer and renal cell carcinoma, where they can induce durable responses and significantly prolong survival (1, 2). In colorectal cancer (CRC), however, this success is largely restricted to the minority of tumors (10-15%) characterized by deficient mismatch repair and high microsatellite instability (dMMR/MSI-H), with a drop to approximately 4–5% in the advanced stage (3).

In dMMR/MSI-H tumors, ICIs produce objective response rates of 30% to 60% with durable clinical benefit, but 13% to 29% of patients exhibit primary progressive disease, and 5% to 25% of initial responders later develop acquired resistance with disease progression (4, 5). The vast majority (85-90%) of tumors are proficient mismatch repair/microsatellite stable (pMMR/MSS) and remain largely unresponsive to ICI monotherapy, showing only modest or mixed benefit from current combination strategies (6–9). The distinct sensitivity of dMMR/MSI-H tumors to immunotherapy is generally attributed to their high mutational burden, increased neoantigen generation, prominent lymphocytic infiltration and unique metabolic features (**Table 1**) (4, 10–12). In contrast, pMMR/MSS CRC are frequently associated with metabolic adaptations that favor immune evasion, characterized by enhanced mitochondrial efficiency and resistance to oxidative stress (**Table 1**) (13–15). These observations suggest that metabolic state, and in particular mitochondrial activity, may contribute to divergent immunotherapy responses across CRC subtypes.

**Table 1:** Biological features associated with differential immunotherapy responses in dMMR/MSI-H and pMMR/MSS colorectal cancer.

Features	dMMR/MSI-H CRC	pMMR/MSS CRC	References
Immune infiltration	Higher frequency of TILs, including CD8^+^ T-cells, NK cells and M1 macrophages.	Frequently, but not uniformly, characterized by reduced CD8^+^ T-cell infiltration, increased Tregs and M2 macrophages, and lower MHC-I expression.	(6, 16)
Antigenicity	High TMB/neoantigen load due to MMR loss, generally preserved antigen presentation and IFN-γ responsiveness.	Generally lower TMB, fewer neoantigens and clonal neoantigens; antigen-presentation defects and impaired IFN-γ signaling may contribute to immune escape.	(6, 17, 18)
Checkpoint expression	High PD-1/PD-L1, CTLA-4 and LAG-3 expression associated with inflamed tumors	Generally lower adaptive immune activation and immune checkpoint expression, although inducible in selected tumors	(5, 16)
Stromal/myeloid barriers	Present, but generally insufficient to restrain antitumor inflammation	Enriched in MDSCs, Tregs, TAMs and dense stromal barriers, frequently associated with WNT/β-catenin- and TGF-β-mediated immune exclusion.	(17, 19)
Hypoxia/lactate	Inflamed tumors may acquire metabolic adaptations associated with acquired resistance	Hypoxia and lactate stabilize suppression and immune escape.	(20, 21)
OXPHOS/mtDNA	Some studies suggest lower mtDNA copy number and a preferential reliance on glycolysis.	Higher mtDNA copy number has been associated with enhanced OXPHOS activity and tumor progression in selected studies.	(22)
Potential therapeutic vulnerabilities	Immune checkpoint blockade (PD-1 ± CTLA-4); mechanisms of primary and acquired resistance remain under investigation.	Combination strategies targeting mitochondrial metabolism, stromal remodeling, immune exclusion and immune checkpoints are under active investigation.	

The features summarized in this table represent general biological trends rather than uniform characteristics of all dMMR/MSI-H and pMMR/MSS CRCs. Considerable biological heterogeneity exists according to consensus molecular subtype (CMS), metastatic site and additional molecular alterations. TIL, tumor-infiltrating leukocytes; NK, natural killer; Treg, regulatory T-cells; MHC, major histocompatibily complex; TMB, tumor mutation burden; MMR, mismatched repair; IFN, interferon; PD-1, Programmed Cell Death Protein 1; PD-L1, Programmed Death-Ligand 1; CTLA-4, cytotoxic T-lymphocyte associated protein 4; LAG3, Lymphocyte-Activation Gene 3; MDSC, myeloid-derived suppressor cells; WNT, wingless-related integration site; TGF, Transforming growth factor; mtDNA, mitochondrial DNA.

Recent studies showed that the tumor microenvironment (TME) plays a critical role in the response to immunotherapy, with metabolic constraints emerging as major regulators of anti-tumor immunity (23–25). In particular, hypoxia and mitochondrial dysfunction can contribute to the stabilization of hypoxia-inducible factor-1α (HIF-1α), which enhances the expression of programmed death-ligand 1 (PD-L1) on tumor cells (26, 27). In parallel, mitochondrial reactive oxygen species (ROS) can activate redox-sensitive factors, such as NF-kB and STAT3, further promoting PD-L1 expression and immunosuppressive signaling (28–30). In addition, rapidly proliferating tumor cells exhibit high rates of glucose and amino acid consumption, thus depriving tumor-infiltrating leucocytes of energy sources required for activation and effector functions (31, 32). This nutrient limitation impacts mitochondrial metabolism in T-cells, as well as in NK cells and antigen-presenting cells, thus restricting glycolytic reprogramming and oxidative phosphorylation (33–36).

In this review, we summarize current insights into mitochondrial regulation of tumor immune escape and immune cell dysfunction in CRC. We further discuss emerging therapeutic strategies aimed at targeting mitochondrial metabolism in both tumor and immune cells that may help overcome resistance and improve responses to immunotherapy. Throughout this review, we propose the concept of the mitochondrial checkpoint as an integrative model describing how multiple mitochondrial-dependent processes operating across tumor cells, immune cells and the tumor microenvironment collectively shape antitumor immunity and responsiveness to immunotherapy. Importantly, this model does not represent a discrete molecular checkpoint analogous to PD-1 or CTLA-4 signaling, but rather a context-dependent view of mitochondrial regulation in cancer.

## 2 Mitochondrial dysfunction and immune evasion

Mitochondrial dysfunction is now recognized as one of the major determinants of tumor immune escape, due to its ability to trigger metabolic rewiring, release of mitochondrial danger signals and modulation of immune checkpoint pathways. In CRC, mitochondrial alterations are highly prevalent and contribute to tumor progression. In particular, mitochondrial DNA (mtDNA) mutations can occur to different extents in primary and metastatic CRC lesions, suggesting that mitochondrial dysfunction may evolve during tumor progression and metastatic dissemination (22, 37).

### 2.1 Tumor cell mitochondrial alterations drive immune activation

One of the most studied consequences of tumor mitochondrial dysfunction is the release of mitochondrial-derived danger-associated molecular patterns (DAMPs), particularly mtDNA. Under acute mitochondrial stress, mtDNA leakage into the cytosol can activate the cGAS-STING pathway and promote anti-tumor immunity (38, 39). In CRC, Gasdermin E-mediated mitochondrial damage was shown to induce mtDNA release, which can trigger cGAS-STING signaling, leading to type I interferon (IFN) production and increased infiltration of cytotoxic CD8^+^ cells (40). Similarly, short-chain acyl-CoA dehydrogenase (ACADS) was recently reported to regulate the demethylation and cytosolic release of mtDNA, thus promoting anti-tumor response in CRC (41). In addition, depletion of serine in CRC cells is able to induce mitochondrial perturbations that trigger cytosolic mtDNA accumulation and subsequent activation of cGAS-STING signaling, stimulating tumor-targeting immune responses (39). Moreover, cGAS-STING signaling and anti-tumor immunity in CRC cells may be triggered by the NLRP3 inflammasome through a positive feedback loop (42), while IL-17A-induced mitochondrial dysfunction can promote cell death by pyroptosis in CRC cells and CD8^+^ T-cell infiltration, further supporting the immunogenic potential of mitochondrial damage (43).

### 2.2 Chronic mitochondrial dysfunction promotes immune escape

Although acute mitochondrial perturbations can activate innate immune pathways and promote anti-tumor immunity, persistent mitochondrial dysfunction often has the opposite effect. Under chronic stress conditions, sustained mitochondrial alterations promote immune checkpoint expression and the establishment of an immunosuppressive tumor microenvironment, ultimately facilitating immune escape. Thus, the biological consequences of mitochondrial dysfunction depend not only on its nature but also on its duration, severity and cellular context. Several mechanisms have been proposed through which chronic mitochondrial dysfunction may promote immune escape. However, not all of these mechanisms have been directly demonstrated in CRC, and some are primarily supported by studies performed in other tumor types. For example, Cheng et al. demonstrated in oral squamous cell carcinoma that mitochondrial Lon protease dysfunction induces mtDNA leakage, leading to PD-L1 upregulation through STING-IFN signaling and extracellular vesicle release, thereby promoting immune escape (29). Although this specific pathway has not yet been demonstrated in CRC, LonP1 is overexpressed in CRC tissues and cell lines, where it promotes glycolytic reprogramming and epithelial-mesenchymal transition, suggesting that LonP1-mediated mitochondrial dysfunction may contribute to CRC progression (44). A second mechanism through which chronic mitochondrial dysfunction promotes immune escape involves metabolic reprogramming of the tumor microenvironment. For example, mitochondrial alterations may associate with compensatory glycolytic activation, leading to lactate accumulation, acidification and metabolic competition within the TME, which impair effector T-cell function and promote immunosuppressive myeloid and Treg populations (45–47). In MSI-H CRC, integrated single-cell and spatial transcriptomic analyses revealed that glycolysis-driven metabolic rewiring is associated with suppressive immune niches and reduced anti-tumor immunity (48). Intercellular mitochondrial communication may represent an additional mechanism linking mitochondrial dysfunction to immune escape. However, direct evidence supporting mitochondrial transfer from CRC cells to immune cells remains limited, and current knowledge is largely extrapolated from other tumor types (49).

### 2.3 Mitochondrial dysfunction and immune cell exhaustion

In order to survive in the nutrient-deprived and highly suppressive TME, tumor-infiltrating immune cells, particularly T-cells, must remain metabolically flexible and preserve mitochondrial fitness. However, these cells frequently exhibit impaired oxidative phosphorylation, altered mitochondrial mass and membrane potential, and defective mitochondrial biogenesis. For example, it was reported that tumor-infiltrating T-cells showed a progressive loss of peroxisome proliferator-activated receptor γ coactivator-1α (PGC1-α), which programs mitochondrial biogenesis, induced by chronic Akt signaling in tumor-specific T-cells, leading to metabolic insufficiency and impaired effector function (35). These alterations appear early during T-cell dysfunction, as immune checkpoint signaling itself contributes to mitochondrial dysregulation. In particular, PD-1 signaling has been associated with bioenergetic insufficiency, reduced mitochondrial respiration and a progressive loss of T-cell functionality during the early stages of exhaustion (50). Chronic antigen exposure exacerbates this exhausted phenotype, as impaired mitochondrial oxidative phosphorylation limits the self-renewal and proliferative capacity of T-cells, thereby limiting long-term anti-tumor immunity (51). Importantly, T-cell exhaustion comprises distinct differentiation states associated with progressive mitochondrial dysfunction. Notably, the expression of inhibitory receptors alone is insufficient to define an exhausted T-cell state and should be interpreted together with functional impairment, differentiation status and exhaustion-associated transcriptional programs. Accordingly, TCF1-positive progenitor-exhausted CD8+ T-cells retain self-renewal capacity and responsiveness to PD-1 blockade, whereas terminally exhausted CD8+ T-cells exhibit sustained expression of inhibitory receptors, including PD-1, TIM-3 and LAG-3, together with impaired effector function and TOX- and NR4A-driven transcriptional programs. Transition toward terminal exhaustion is accompanied by mitochondrial depolarization, reduced spare respiratory capacity and impaired mitophagy, resulting in the accumulation of dysfunctional mitochondria and increased oxidative stress (41, 52). Similar metabolic defects have also been observed in human solid tumors, such as renal cell carcinoma, where tumor-infiltrating CD8^+^ T-cells exhibit both mitochondrial dysfunction and glycolytic insufficiency, suggesting that metabolic collapse of immune cells may be a conserved hallmark of dysfunctional anti-tumor immunity across cancer types (53). Beyond maintaining bioenergetic homeostasis, mitochondrial metabolism and redox balance are essential for T-cell activation, differentiation and effector function (54–56). For example, mitochondrial reactive oxygen species (mtROS) are required for proper antigen-specific T-cell activation, NFAT activation and IL-2 induction, as T-cells with reduced mtROS had normal bioenergetics, but failed to expand in response to antigen (55). Thus, while transient and controlled mtROS production acts as a physiological signaling mechanism during T-cell activation, chronic antigenic stimulation and metabolic stress within the TME can lead to persistent ROS accumulation beyond physiological signaling levels. This sustained oxidative stress reinforces NFAT-dependent TOX- and NR4A-mediated exhaustion programs, ultimately contributing to T-cell dysfunction (41). These observations indicate that therapeutic strategies aimed at restoring mitochondrial fitness in exhausted T-cells may represent a valuable approach to improve responsiveness to immunotherapy. Collectively, these observations suggest that therapeutic strategies aimed at restoring mitochondrial fitness may improve T-cell function and enhance responsiveness to immunotherapy. Consistent with this concept, recent studies further identify impaired mitochondrial biogenesis, dynamics and mitophagy as interconnected drivers of T-cell exhaustion across cancer and chronic infection settings (57).

Although most current evidence derives from non-CRC tumor models, mitochondrial dysfunction may also influence the activity of other immune cells, including natural killer (NK) cells. In particular, altered mitochondrial dynamics, such as excessive mitochondrial fragmentation, were reported to impair NK-cell persistence and limit memory formation in liver cancer, ultimately reducing anti-tumor activity (58). In parallel, tumor-derived suppressive molecules, such as TGF-β, can directly affect mitochondrial metabolism in NK cells in breast cancer, leading to reduced cytotoxic capacity and functional exhaustion (59). However, comparable mechanistic studies in CRC are still lacking.

Myeloid populations are increasingly recognized as key regulators of the immunosuppressive tumor microenvironment and are profoundly influenced by mitochondrial rewiring. Disruption of mitochondrial quality control and chaperone function in macrophages has been linked to immunosuppressive macrophage activity and tumor immune escape in melanoma, highlighting the role of mitochondrial homeostasis in maintaining anti-tumor immunity (60). Moreover, metabolic rewiring through the SENP1-SIRT3 axis increases acetyl-CoA in macrophage mitochondria, which promotes cholesterol biosynthesis, thus reinforcing immunosuppressive phenotypes and limiting effective anti-tumor responses (61). In addition, mitochondrial regulation in macrophages has recently been linked to immune infiltration, as P2X4 receptor-dependent signaling in M1-like tumor-associated macrophages induces mitochondrial dysfunction and M1-like polarization, thereby regulating T-cell infiltration and function within the CRC microenvironment (62).

Despite increasing interest in immune metabolism, specific evidence in CRC patients remains limited. In rectal cancer patients at high risk of metastatic progression, the constitutional mtDNA status in blood was linked to systemic T-cell immunity and metastatic susceptibility, with numerous mtDNA variants associated with expanded helper and cytotoxic T-cell populations and a reduced risk of metastasis, whereas higher levels of damaged cell-free mtDNA in serum correlated with pro-tumorigenic inflammation (63). More recently, mtDNA copy number was shown to have opposing relationships with T-cell infiltration and prognosis in dMMR/MSI-H versus pMMR/MSS CRC, indicating that mitochondrial regulation may differ substantially across molecular CRC subtypes (64).

Overall, although mechanistic studies performed in melanoma, renal cancer, breast cancer and other solid tumors have provided important insights into the role of mitochondrial dysfunction in immune regulation, direct evidence supporting these mechanisms in CRC remains comparatively limited. Therefore, findings extrapolated from other tumor types should be interpreted cautiously until validated in CRC-specific experimental and clinical settings.

### 2.4 Tumor microenvironment as a driver of mitochondrial dysfunction and immune suppression

TME is known to impact the response to immunotherapy. For example, TME in MSS CRC patients is characterized by the presence of several metabolic stressors that can drive mitochondrial dysfunction in the immune cells, while sparing or even supporting mitochondrial fitness in tumor cells (65, 66). This imbalance creates a permissive niche for tumor persistence and a major barrier to ICIs. One of the main features of the TME is the competition for key metabolic resources. Rapidly proliferating tumor cells exhibit high rates of glucose and amino acid consumption and were reported to be able to deprive tumor-infiltrating leukocytes of the substrates required for their activation and effector function (31). For example, this nutrient limitation directly impacts mitochondrial metabolism in T-cells, restricting both glycolytic reprogramming and oxidative phosphorylation (31, 33, 53, 67). Indeed, T-cells fail to sustain ATP production and biosynthesis in glucose-depleted conditions, leading to impaired mitochondrial function and reduced cytokine production (68). Similarly, depletion of amino acids, such as glutamine and arginine, affects mitochondrial anaplerosis and metabolic flexibility (69, 70). In contrast, tumor cells often maintain access to alternative metabolic pathways, reinforcing their survival over immune activity (71).

Hypoxia is also another important hallmark of the CRC microenvironment and a major determinant of mitochondrial function. Stabilization of hypoxia-inducible factors (HIFs) can induce transcriptional programs that suppress mitochondrial respiration in immune cells while promoting adaptive responses in tumor cells (72). For example, hypoxia impairs mitochondrial biogenesis and electron transport chain activity in T lymphocytes, limiting their ability to generate energy and sustain effector functions (73, 74). Hypoxia may also promote the expression of inhibitory receptors and contribute to T-cell exhaustion in nasopharyngeal carcinoma (74). In contrast, tumor cells are able to adapt to hypoxic conditions in prostate adenocarcinoma and glioblastoma by inducing mitochondrial reprogramming that preserves redox balance and supports survival (75).

Interestingly, the metabolic activity of tumor cells leads to the accumulation of byproducts in the TME that profoundly affect mitochondrial function in immune cells. For example, lactate, a major product of tumor glycolysis, accumulates in the TME and can inhibit T-cell activation and cytokine production (45, 76–78). Elevated lactate levels also disrupt mitochondrial metabolism by altering intracellular pH and inhibiting key enzymatic processes (76). Other metabolites, such as succinate and itaconate, can also modulate immune cell function through effects on mitochondrial signaling pathways and epigenetic regulation, as they were reported to contribute to the polarization of macrophages toward immunosuppressive phenotypes (79, 80).

Non-malignant components of the TME, including cancer-associated fibroblasts and mesenchymal stromal cells, play an active role in shaping mitochondrial dynamics. These cells can provide metabolic substrates (such as lactate, fatty acids and glutamine), modulate redox balance and influence mitochondrial function in both tumor and immune cells (81–83). Moreover, emerging evidence suggests that stromal cells may also contribute to intercellular mitochondrial transfer, particularly toward tumor cells, thereby enhancing their bioenergetic capacity and resistance to stress (84). While the direct impact of such processes on immunotherapy response in CRC remains to be fully defined, stromal-tumor interactions likely contribute to the maintenance of mitochondrial fitness in cancer cells under immune pressure.

In conclusion, TME is not merely a passive context, but an active driver of mitochondrial dysfunction and immune suppression in CRC. By orchestrating metabolic constraints that differentially impact tumor and immune cells, the TME reinforces resistance to immunotherapy.

Based on the evidence discussed above, we propose the term “mitochondrial checkpoint” to describe the integrated network of mitochondrial-dependent processes operating across tumor cells, immune cells and the tumor microenvironment that collectively regulate anti-tumor immunity and responsiveness to immunotherapy in CRC. Importantly, we do not propose the mitochondrial checkpoint as an established molecular checkpoint analogous to PD-1/PD-L1 or CTLA-4 signaling. Rather, we introduce it as a conceptual framework encompassing multiple interconnected mitochondrial-dependent processes that collectively regulate antitumor immunity. Within this framework, mitochondrial adaptations may exert distinct or even opposing effects depending on the cellular compartment, the duration of stress and the surrounding microenvironment.

## 3 Mitochondrial plasticity and immunotherapy resistance

As outlined above, the distinct clinical responsiveness of dMMR/MSI-H and pMMR/MSS CRC to ICIs is thought to reflect, at least in part, differences in mitochondrial function and metabolic adaptation. Since dMMR/MSI-H tumors are already largely responsive to ICI monotherapy, the mitochondrial resistance mechanisms discussed below, several of which have been characterized in CRC models not specifically stratified by MSI status, are of particular relevance to pMMR/MSS CRC, where overcoming resistance to immunotherapy remains an unmet clinical need.

Mitochondria can sustain tumor cells, including CRC cells, to efficiently adapt in response to environmental and therapeutic stress, making them able to maintain metabolic homeostasis, resist immune-mediated damage and survive under strict conditions of TME, thus contributing to both primary and acquired resistance to ICIs (**Figure 1**). In particular, CRC cells can easily switch their metabolic preferences from glycolysis to oxidative phosphorylation (OXPHOS) and vice versa, according to oxygen and nutrient availability (85). For example, tumor cells can shift toward mitochondrial oxidative phosphorylation in response to stress within the tumor microenvironment, thereby sustaining ATP production and activating survival pathways, including under pressure from cytotoxic immune cells and interferon signaling (86, 87). All these actions significantly contribute to the maintenance of energy production under nutrient-limited conditions and protection in response to oxidative and metabolic stress, thus avoiding metabolic vulnerabilities that might otherwise be exploited by immune cells. As a result, mitochondrial plasticity reduces the effectiveness of immune-mediated killing and contributes to resistance to ICIs. Another mechanism by which mitochondria can contribute to resistance to immunotherapy is mitophagy, which maintains their integrity and function by a selective degradation of damaged mitochondria (88). Indeed, during metabolic stress and immune-mediated attack, increased ROS production and metabolic disruption can damage mitochondrial components, but mitophagy can be triggered by different pathways, such as PINK1/Parkin signaling, thus eliminating dysfunctional mitochondria in tumor cells, preventing the accumulation of toxic byproducts and avoiding apoptosis (89). Consistent with this adaptive role, recent evidence indicates that mitochondrial fission and mitophagy exert a context-dependent, dual influence on tumor cell survival, since excessive fission can trigger apoptosis, whereas in most cancer settings, including CRC, coordinated fission-mitophagy cycles instead sustain mitochondrial fitness and confer resistance to cell death (90, 91). CRC cells can also actively regenerate mitochondrial populations through mitochondrial biogenesis, a process regulated by the transcriptional coactivators such as PGC-1α and downstream factors, including NRF1 and TFAM, which coordinate the replication of mtDNA and the synthesis of mitochondrial proteins (92, 93). This mechanism significantly contributes to energy restoration following immune-mediated damage, long-term tumor survival and resistance to repeated cycles of immune attack, allowing tumor cells to recover from transient immune responses.

**Figure 1:**
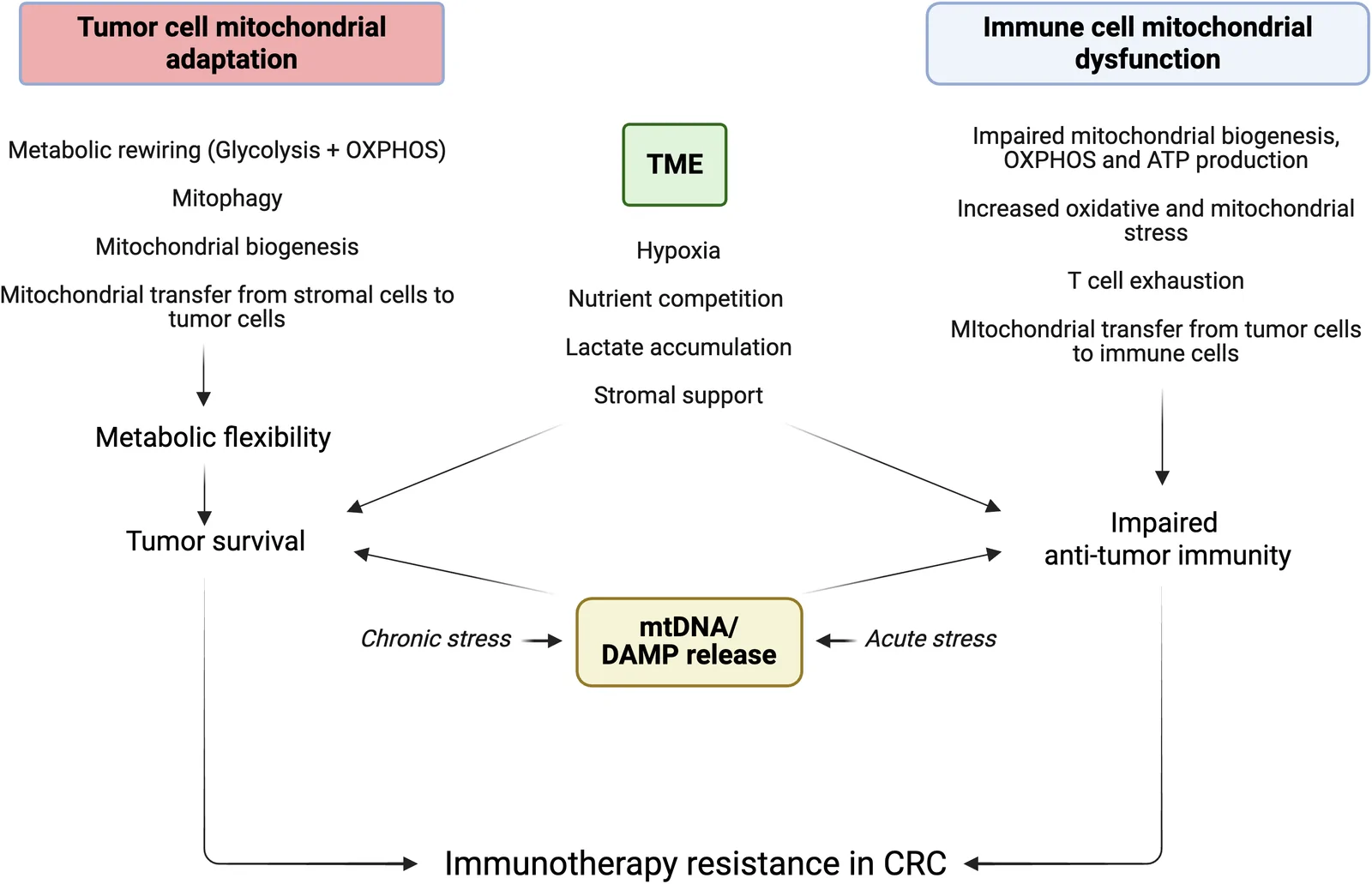
Mitochondrial mechanisms underlying immunotherapy resistance in CRC. Schematic overview of the mitochondrial mechanisms contributing to immunotherapy resistance in colorectal cancer (CRC). On the left, tumor cells undergo mitochondrial adaptation through metabolic rewiring, increased mitophagy, mitochondrial biogenesis, and mitochondrial transfer from stromal cells, collectively enhancing metabolic flexibility and promoting tumor survival. On the right, immune cells develop mitochondrial dysfunction characterized by impaired mitochondrial biogenesis, reduced OXPHOS and ATP production, increased oxidative and mitochondrial stress, T-cell exhaustion, and potential mitochondrial transfer from tumor cells, leading to impaired antitumor immunity. The tumor microenvironment (TME), through hypoxia, nutrient competition, lactate accumulation, and stromal support, further influences both tumor cell adaptation and immune cell dysfunction. In addition, mitochondrial danger signaling mediated by mtDNA/DAMP release represents an integral component of the proposed mitochondrial checkpoint, linking acute or chronic mitochondrial stress to the regulation of tumor survival and antitumor immune responses. Together, these interconnected mechanisms contribute to immunotherapy resistance in CRC. ATP, adenosine triphosphate; CRC, colorectal cancer; DAMP, damage-associated molecular pattern; mtDNA, mitochondrial DNA; OXPHOS, oxidative phosphorylation; TME, tumor microenvironment. Created in https://BioRender.com.

In conclusion, metabolic rewiring, mitophagy and mitochondrial biogenesis represent an integrated adaptive network that enables CRC cells to respond dynamically to immune pressure and to maintain mitochondrial function despite environmental stress, creating a moving target for immune-mediated elimination. Importantly, this adaptive capacity contrasts with the limited plasticity observed in exhausted immune cells, which often fail to restore mitochondrial function. This imbalance further reinforces the role of mitochondria in immunotherapy resistance, where tumor cells retain adaptability while immune cells remain metabolically disadvantaged. Moreover, whether these adaptive mechanisms differ quantitatively or qualitatively between dMMR/MSI-H and pMMR/MSS CRC remains largely unexplored and represents an important area for future investigation.

## 4 Intercellular mitochondrial transfer and modulation of anti-tumor immunity

Although intercellular mitochondrial transfer has emerged as an important mechanism of metabolic communication within the tumor microenvironment, direct evidence linking this process to immunotherapy resistance in CRC remains limited. Current knowledge derives predominantly from studies investigating mitochondrial transfer from stromal cells to CRC cells, whereas evidence regarding mitochondrial transfer involving immune cells or extracellular vesicle (EV)-mediated mtDNA signaling is more limited and often extrapolated from other tumor types. Because these mechanisms differ substantially in their biological nature and functional consequences, they should be considered separately.

Transfer of intact mitochondria from stromal cells to CRC cells represents the best-characterized mechanism in CRC. CRC cells can receive functional mitochondria from surrounding stromal cells, particularly bone-marrow mesenchymal stem cells (BM-MSCs), through tunneling nanotubes (TNTs). Under 5-fluorouracil (5-FU)-induced stress, CRC cells increase secretion of oxidized High Mobility Group Box 1 (HMGB1), which stimulates BM-MSCs to extend TNTs and transfer mitochondria into tumor cells (84). This process restores mitochondrial membrane potential, reduces mitochondrial ROS accumulation and is associated with enhanced cancer cell stemness and reduced sensitivity to 5-FU *in vitro* and in murine CRC models (84). These findings indicate that stromal-to-tumor mitochondrial transfer enhances mitochondrial fitness and metabolic adaptability of CRC cells under therapeutic stress.

Distinct from the transfer of intact organelles, CRC cells can also release mitochondrial components through EVs. In CRC patients, serum-derived EVs enriched in circular mtDNA deliver their cargo to neighboring normal colonic epithelial cells, enhancing oxidative phosphorylation, ROS production and TGF-β1 signaling, thereby promoting pro-tumorigenic metabolic reprogramming (94). Although this process does not involve the transfer of functional mitochondria, it demonstrates that mitochondrial genetic material can act as an intercellular signaling molecule capable of modifying the metabolic behavior of recipient cells.

Whether direct mitochondrial transfer occurs between CRC cells and immune cells remains largely unknown. Nevertheless, studies in other tumor types suggest that mitochondrial exchange may influence immune-cell metabolism and function, raising the possibility that mitochondrial transfer may also contribute to immune dysfunction and resistance to immunotherapy. At present, however, direct experimental evidence supporting this mechanism in CRC is lacking, and further studies are required to determine whether bidirectional mitochondrial transfer between tumor and immune cells occurs in CRC *in vivo* and whether it has functional consequences for immunotherapy responsiveness.

Collectively, these observations suggest that intercellular mitochondrial communication represents an emerging regulator of tumor metabolic plasticity and tumor-host interactions. However, the biological significance of stromal-to-tumor mitochondrial transfer, extracellular mtDNA signaling and potential tumor-immune mitochondrial exchange should be considered distinct processes, each requiring further mechanistic investigation before their contribution to immunotherapy resistance in CRC can be established.

## 5 Therapeutic targeting of mitochondrial pathways and biomarkers of immunotherapy response

Since mitochondrial dysfunction can deeply influence the response to immunotherapy, it may represent a valuable therapeutic target in CRC treatment. By modulating mitochondrial pathways in both tumor and immune cells, it may be possible to weaken tumor mitochondrial adaptability while simultaneously restoring mitochondrial fitness in immune cells, thus overcoming resistance and enhancing the efficacy of ICIs (**Figure 2**). Given that dMMR/MSI-H CRC already obtains substantial benefit from ICI monotherapy, these mitochondria-targeted strategies are expected to be most clinically relevant for pMMR/MSS CRC and for the subset of dMMR/MSI-H tumors with primary or acquired ICI resistance.

**Figure 2:**
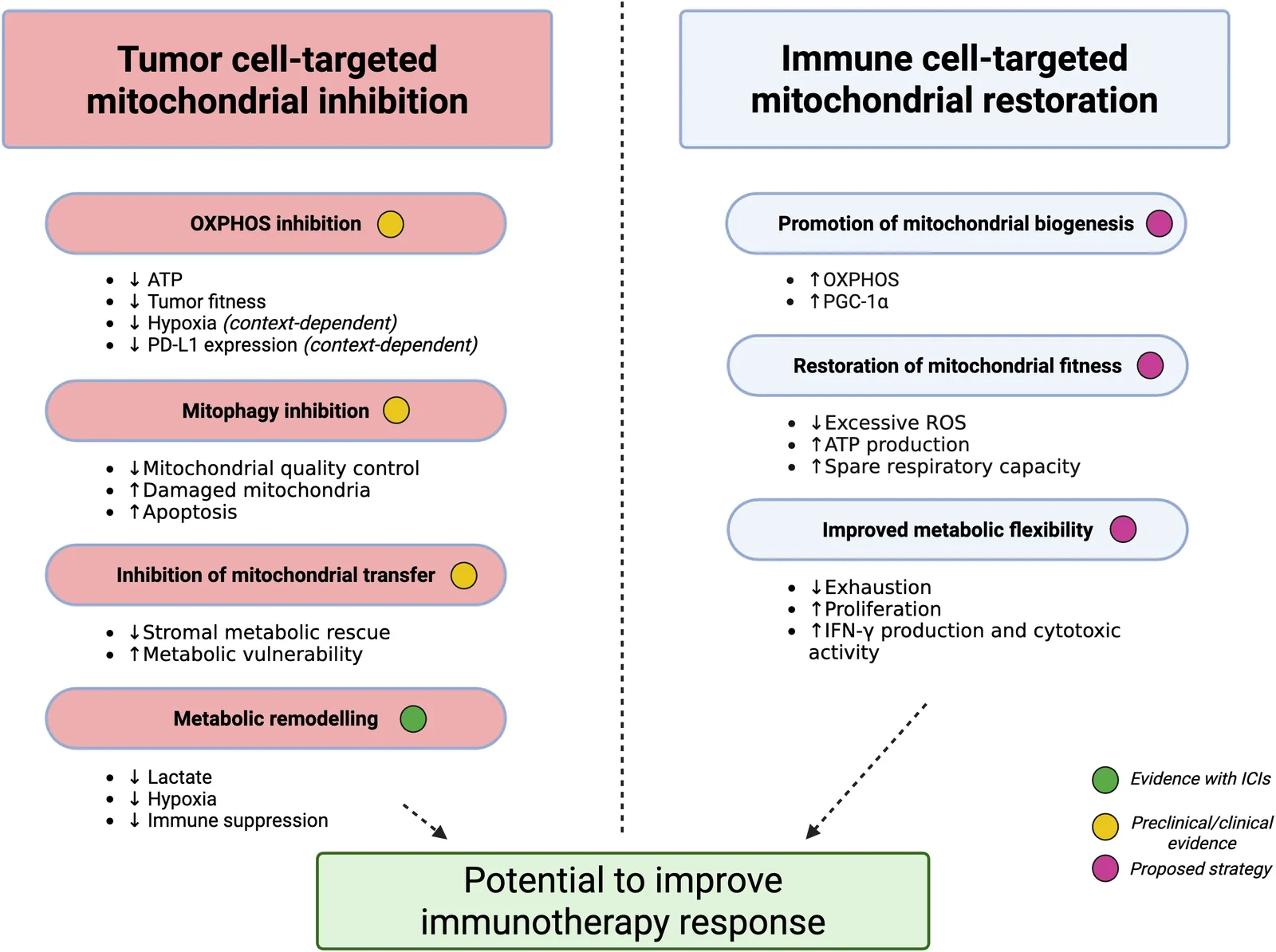
Therapeutic strategies targeting mitochondrial pathways with the potential to enhance immunotherapy efficacy in CRC. Schematic representation of therapeutic approaches targeting mitochondrial pathways with the potential to improve immunotherapy responses in CRC. Left panel: tumor cell-directed interventions aim to reduce tumor metabolic fitness by inhibiting OXPHOS, blocking mitophagy, preventing mitochondrial transfer from stromal cells, and remodeling tumor metabolism, thereby increasing metabolic vulnerability, promoting apoptosis, and alleviating hypoxia- and lactate-driven immunosuppression. The effects of OXPHOS inhibition on hypoxia and PD-L1 expression are context-dependent. Right panel: immune cell-directed strategies focus on restoring mitochondrial function by promoting mitochondrial biogenesis, improving mitochondrial fitness, and enhancing metabolic flexibility. These interventions increase OXPHOS, ATP production, spare respiratory capacity, proliferation, and IFN-γ-mediated cytotoxic activity while reducing oxidative stress and T-cell exhaustion. Colored symbols indicate the current level of evidence supporting each strategy: green, evidence in combination with immune checkpoint inhibitors (ICIs); yellow, preclinical or clinical evidence supporting antitumor or metabolic effects; purple, proposed strategies with limited direct evidence. Dashed arrows indicate the potential, rather than established, contribution of these approaches to improving immunotherapy responses in CRC. ATP, adenosine triphosphate; CRC, colorectal cancer; ICIs, immune checkpoint inhibitors; IFN-γ, interferon gamma; OXPHOS, oxidative phosphorylation; PD-L1, programmed death-ligand 1; PGC-1α, peroxisome proliferator-activated receptor gamma coactivator 1-alpha; ROS, reactive oxygen species. Created in https://BioRender.com.

Several agents, including metformin and other experimental complex I inhibitors, can impair mitochondrial respiration by targeting complex I activity, thereby reducing ATP production, activating AMPK-dependent metabolic stress responses and limiting tumor cell proliferation and metabolic fitness (**Table 2**) (95, 96). Notably, metformin has been associated with improved progression-free survival when combined with first-line systemic therapy in patients with metastatic CRC, supporting further investigation of therapies targeting this respiratory complex as an adjuvant strategy to enhance anticancer therapies (97). Moreover, metformin administration has been shown to reduce the incidence of metachronous colorectal adenomas by approximately one-third in non-diabetic patients following polypectomy, suggesting that mitochondrial targeting may also have preventive potential in colorectal carcinogenesis. Moreover, preclinical studies have shown that metformin combined with the glycolysis inhibitor 2-deoxyglucose (2-DG) decreases mitochondrial membrane potential and suppresses CRC cell invasiveness (98).

**Table 2:** Translational overview of mitochondria-targeted therapeutic strategies in colorectal cancer.

Therapeutic strategy	Key findings	Evidence level	Experimental model	Potential biomarker(s)	Immunotherapy combination	Limitations/concerns
OXPHOS inhibition (VLX600)	VLX600 inhibits mitochondrial respiration by targeting Complex I, II and IV, thereby reducing oxygen consumption and inducing mitochondrial dysfunction in CRC cells (99).	Preclinical (*in vitro* + immunodeficient xenograft)	HCT116 and HT29 xenograft models	OXPHOS signature; OCR (potential predictive)	Not evaluated	Immunodeficient xenograft; effects on antitumor immunity, immune-cell metabolism and intestinal homeostasis remain unknown.
OXPHOS inhibition (ASP4132)	ASP4132 inhibits mitochondrial Complex I, as demonstrated by increased serum lactate levels, but showed limited clinical activity due to dose-limiting toxicities (100).	Phase I clinical trial	Patients with treatment-refractory advanced solid tumors.	Serum lactate (pharmacodynamic biomarker)	Not evaluated.	Limited clinical activity; dose-limiting toxicities prevented further dose escalation.
OXPHOS inhibition (IACS-010759)	Compound IACS-010759 inhibits mitochondrial Complex I, causing aspartate depletion, replication stress and reduced CRC growth (101).	Preclinical (*in vitro* + immunodeficient xenograft + patient-derived xenograft)	CRC cell lines (HCT116, Colo205, LIM1215, Caco-2, RKO, LS411N, SW620), NSG xenografts and CRC patient-derived xenografts (PDXs).	OCR, aspartate levels, replication stress markers (potential predictive); GOT1 expression (potential predictive).	Not evaluated.	Evidence derives from immunodeficient models; no assessment of anti-tumor immunity or immunotherapy combinations.
OXPHOS modulation (SIRT1/PGC-1α inhibition)	Inhibition of the SIRT1/PGC-1α axis suppresses OXPHOS reprogramming and restores chemosensitivity in patient-derived CRC models and xenografts (102).	Preclinical (patient-derived models + immunodeficient xenograft)	Chemotherapy-treated CRC liver metastases, patient-derived colonospheres, and nude mouse xenografts.	SIRT1, PGC-1α, OXPHOS gene signature, COXIV expression, OCR	Not evaluated	Focused on chemotherapy resistance rather than immunotherapy; preclinical validation only; no pharmacological SIRT1/PGC-1α-targeted clinical evaluation.
OXPHOS inhibition (Metformin + 2-DG)	Reverses radioresistance by targeting mitochondrial metabolism, reducing T-cell exhaustion and enhancing radiotherapy efficacy (98).	Preclinical (*in vitro* + patient samples + immunocompetent syngeneic model)	CT26 CRC cells; immunocompetent CT26 tumor-bearing BALB/c mice; clinical CRC samples.	p-eIF2α/ATF4 expression; T-cell exhaustion markers (PD-1, TIM-3); mitochondrial stress response (potential predictive).	Not evaluated (combined with radiotherapy only).	Evidence derives from radiotherapy models rather than ICI therapy; metformin may also affect immune-cell metabolism, requiring careful dose optimization.
OXPHOS inhibition (Metformin)	Direct inhibition of mitochondrial complex I suppresses oxidative phosphorylation, reduces HIF-1 activation and tumor growth through cancer cell-autonomous mechanisms (103).	Preclinical (*in vitro* + immunodeficient xenograft)	Human CRC cell lines (HCT116), lung cancer cells (A549), xenograft mouse models.	Complex I activity; HIF-1α; VEGF; CA9; OCT transporter expression (potential predictor of metformin sensitivity).	Not evaluated.	No immunotherapy experiments; immunodeficient xenograft models; mechanism demonstrated mainly in tumor cells.
OXPHOS inhibition (Metformin; systemic therapy)	Associated with improved progression-free survival, but not overall survival, in patients with metastatic CRC receiving first-line systemic therapy (97).	Retrospective clinical study	Patients with metastatic CRC receiving first-line fluoropyrimidine-based chemotherapy plus anti-EGFR or bevacizumab.	No validated biomarker	Not evaluated.	Retrospective single-center study; all metformin users were diabetic; association only; no mechanistic evaluation; no immunotherapy.
OXPHOS inhibition (Metformin; chemoprevention)	Low-dose metformin (250 mg/day for 1 year) reduced the incidence of metachronous colorectal adenomas/polyps after endoscopic polypectomy in non-diabetic patients (104).	Prospective randomized phase III clinical trial	Non-diabetic post-polypectomy patients at high risk of adenoma recurrence.	HOMA-IR (potential predictive biomarker); AMPK activation (mechanistic).	Not evaluated.	Chemoprevention setting rather than established CRC; no immunotherapy assessment; 1-year follow-up; benefit on adenoma recurrence rather than cancer outcomes. Low-dose metformin was well tolerated, with only grade 1 adverse events and no serious treatment-related toxicity.
OXPHOS/Glycolysis inhibition (Phenformin + oxamate)	Synergistic inhibition of CRC growth through simultaneous targeting of mitochondrial complex I and LDH, leading to increased ROS, ATP depletion, apoptosis, and reduced tumor growth *in vivo* (105).	Preclinical (*in vitro* + immunocompetent syngeneic model)	CT26 murine CRC cells and immunocompetent BALB/c syngeneic mice; additional validation in multiple cancer cell lines.	Complex I activity, LDH activity, ROS, lactate production, FDG uptake (pharmacodynamic biomarkers).	Not evaluated.	No immunotherapy assessment; phenformin is no longer routinely used clinically because of the risk of lactic acidosis; findings remain preclinical despite efficacy in syngeneic CRC models.
Glycolysis inhibition (LDH-A inhibition + PD-1 blockade)	LDH-A inhibition reduced glycolysis, downregulated MMR proteins and stemness markers (Oct4), suppressed tumor growth, and enhanced the antitumor efficacy of PD-1 blockade in pMMR CRC xenograft models (106).	Preclinical (human tissues + *in vitro* + immunodeficient xenograft)	HCT116 (dMMR), SW480 (pMMR), subcutaneous xenograft models, and analysis of 186 human CRC specimens.	LDH-A expression, MMR proteins (MSH2, MLH1, PMS2/MSH6), Oct4, VEGF, dMMR/pMMR status	Yes – anti-PD-1 (preclinical). LDH-A knockdown potentiated PD-1 blockade in pMMR CRC xenografts	Mechanistic conclusions are based on genetic LDH-A knockdown rather than clinically validated LDH-A inhibitors; only xenograft models were used; no clinical validation of the proposed combination strategy.
Hypoxia pathway inhibition (Dual HIF-1/HIF-2 inhibition + ICB)	Novel dual HIF-1/2 inhibitors (1.21S9N and 3.2.16) disrupted HIF-1/2α-HIF-1β dimerization, induced proteasomal degradation of HIF-1α/HIF-2α, reduced angiogenesis and immunosuppressive signaling, inhibited tumor growth, and markedly enhanced the efficacy of immune checkpoint blockade across several tumor models, including CRC (107).	Preclinical (*in vitro* + immunocompetent and immunodeficient mouse models)	Human CRC (HCT116), murine CRC (CT26, MC38), plus breast cancer, melanoma, HNSCC and prostate cancer models.	HIF-1α, HIF-2α, VEGF, ANGPTL4, PD-L1, CD73, CD47, CA9, LAG-3, TIM-3, PD-1, tumor hypoxia/HIF activity.	Yes. Combination with anti-CTLA-4 produced durable complete responses in MC38 CRC; combination with anti-PD-1 improved efficacy in CT26 CRC and outperformed the selective HIF-2 inhibitor PT2385.	Entirely preclinical; novel HIF inhibitors (1.21S9N and 3.2.16) are not yet clinically available; efficacy demonstrated only in murine models despite broad validation across multiple tumor types.
Amino acid metabolism (ARG2 inhibition)	ARG2 inhibition restored extracellular L-arginine availability, rescued CD8^+^ T-cell glycolysis and proliferation, and synergized with immune checkpoint blockade to overcome ICB resistance in CRC (108).	Preclinical (metabolomics + *in vitro* + immunocompetent murine CRC models)	CRC cell lines, metabolomic analyses, CD8^+^ T-cell co-culture and murine CRC models.	ALDH2, PBX3, ARG2 expression; extracellular L-arginine levels (potential predictive biomarkers).	Yes – anti-PD-1 (preclinical).	Preclinical evidence only; long-term effects of systemic ARG2 inhibition on normal arginine metabolism and immune homeostasis remain unknown.
Amino acid metabolism (OATD-02)	First-in-human study evaluating the safety, tolerability, pharmacokinetics and preliminary antitumor activity of OATD-02 in advanced/metastatic solid tumors. (NCT05759923).	Phase I clinical trial (ongoing)	Patients with advanced/metastatic CRC, pancreatic, renal and ovarian cancers.	ARG1/ARG2 activity; circulating L-arginine (pharmacodynamic, exploratory).	No (monotherapy). The trial evaluates the potential to enhance future immunotherapy responses but does not include immune checkpoint inhibitors	Ongoing dose-escalation study; no efficacy data available yet; safety and clinical benefit remain to be established.
Amino acid metabolism (DRP-104)	Broad glutamine antagonism remodels tumor metabolism and the TME, enhances T-cell and NK-cell infiltration and function, promotes M1 macrophage polarization, reduces MDSCs, and synergizes with immune checkpoint blockade to improve antitumor responses (109).	Preclinical (multiple immunocompetent syngeneic models)	Multiple murine syngeneic tumor models (non CRC-specific).	Glutamine metabolism signature; MDSCs; CD8^+^ TILs; NK-cell infiltration; M1 macrophage polarization (potential pharmacodynamic biomarkers).	Yes – anti-PD-1/PD-L1 and anti-CTLA-4 (preclinical)	Evidence derives predominantly from non-CRC tumor models; clinical efficacy in CRC remains to be established despite ongoing clinical development.
Mitophagy modulation (piR-823 targeting)	piR-823 inhibition activates PINK1/Parkin-mediated mitophagy, induces mitochondrial dysfunction and apoptosis, and suppresses CRC growth through stabilization of PINK1 (110).	Preclinical (human tissues + *in vitro* + immunodeficient xenograft)	CRC cell lines (HCT116, DLD-1, HT29); HCT116 xenograft; human CRC tissues.	piR-823 expression; PINK1; phospho-Parkin (Ser65); mtDNA copy number; TOM20 (mechanistic biomarkers).	Not evaluated.	No immunotherapy assessment; based mainly on immunodeficient xenografts; therapeutic relevance for anti-tumor immunity remains to be established.
Mitophagy modulation (USP14 inhibition)	USP14 inhibition activates BAG4/PRKN-dependent mitophagy, suppresses tumor growth, and enhances oxaliplatin sensitivity in MSI-H colorectal cancer by promoting Parkin recruitment to damaged mitochondria (111).	Preclinical (human tissues + *in vitro* + immunodeficient xenograft)	MSI-H CRC cell lines (LoVo, SW48); patient-derived MSI-H CRC tissues; BALB/c nude mouse xenografts.	USP14, BAG4, PRKN (Parkin), TOM20, TOM22, LC3-II/I.	Not evaluated.	Restricted to MSI-H CRC; no immunotherapy evaluation; evidence based mainly on immunodeficient xenograft models; Clinical translation is limited by the lack of selective USP14 inhibitors.
Mitophagy modulation (Strigolactone analogue 6)	The stereodefined strigolactone analogue 6 selectively inhibits autophagy/mitophagy in CRC cells by blocking autophagosome–lysosome fusion, inducing accumulation of autophagosomes/mitophagosomes, apoptosis, S-phase arrest, and suppressing tumor growth *in vivo* with low toxicity (112).	Preclinical (*in vitro* + immunodeficient xenograft)	HCT116 and SW620 CRC cell lines; NCM460 normal colonic epithelial cells; HCT116 xenograft mouse model.	LC3-II, p62/SQSTM1, LAMP1, PINK1, phospho-Parkin (Ser65), cleaved caspase-3	Not evaluated.	Preclinical study; molecular target of compound 6 remains unidentified; no immunotherapy or patient-derived models; efficacy demonstrated only in xenografts.
Mitochondrial transfer inhibition (oxidized HMGB1)	Oxidized HMGB1 released by chemotherapy-damaged CRC cells promotes mitochondrial transfer from BM-MSCs to CRC cells through NF-κB activation, increasing stemness, oxidative phosphorylation, chemoresistance, and tumorigenicity. HMGB1 neutralization or NF-κB inhibition suppresses mitochondrial transfer and enhances 5-FU efficacy (84).	Preclinical (*in vitro* + immunocompetent and immunodeficient mouse models)	HT29 and CT26 CRC cell lines; human and mouse bone marrow-derived MSCs; mitoception assays; BALB/c syngeneic and xenograft mouse models; Miro1-knockdown bone marrow transplantation model.	Oxidized HMGB1, RAGE, TLR4, NF-κB, Miro1, Cx43, SOX2, LGR5, CD44, KLF4, mtROS, mitochondrial membrane potential (TMRE).	Not evaluated.	Preclinical study; focuses on chemotherapy-induced mitochondrial transfer rather than immune responses; no patient-derived models or immunotherapy combinations; HMGB1 blockade may also affect physiological tissue repair and MSC functions.

2-DG, 2-deoxy-D-glucose; 5-FU, 5-fluorouracil; ALDH2, aldehyde dehydrogenase 2; AMPK, AMP-activated protein kinase; ANGPTL4, angiopoietin-like 4; ARG1, arginase 1; ARG2, arginase 2; ATF4, activating transcription factor 4; ATP, adenosine triphosphate; BAG4, BCL2-associated athanogene 4; BM-MSCs, bone marrow-derived mesenchymal stem cells; CA9, carbonic anhydrase IX; CD, cluster of differentiation; COXIV, cytochrome c oxidase subunit IV; CRC, colorectal cancer; CTLA-4, cytotoxic T-lymphocyte-associated protein 4; CX43, connexin 43; dMMR, deficient mismatch repair; EGFR, epidermal growth factor receptor; FDG, fluorodeoxyglucose; GOT1, glutamate oxaloacetate transaminase 1; HIF, hypoxia-inducible factor; HNSCC, head and neck squamous cell carcinoma; HOMA-IR, homeostatic model assessment of insulin resistance; ICB, immune checkpoint blockade; ICI, immune checkpoint inhibitor; LDH, lactate dehydrogenase; LDH-A, lactate dehydrogenase A; LGR5, leucine-rich repeat-containing G protein-coupled receptor 5; MDSCs, myeloid-derived suppressor cells; MMR, mismatch repair; MSI-H, microsatellite instability-high; mtDNA, mitochondrial DNA; mtROS, mitochondrial reactive oxygen species; NF-κB, nuclear factor kappa B; NK, natural killer; NSG, NOD scid gamma; OCR, oxygen consumption rate; OCT, organic cation transporter; OXPHOS, oxidative phosphorylation; p-eIF2α, phosphorylated eukaryotic initiation factor 2 alpha; PD-1, programmed cell death protein 1; PD-L1, programmed death-ligand 1; PDX, patient-derived xenograft; PGC-1α, peroxisome proliferator-activated receptor gamma coactivator 1-alpha; piR-823, PIWI-interacting RNA-823; pMMR, proficient mismatch repair; PRKN, parkin RBR E3 ubiquitin protein ligase (Parkin); PMS2, postmeiotic segregation increased 2; RAGE, receptor for advanced glycation end products; ROS, reactive oxygen species; SQSTM1, sequestosome 1; SIRT1, sirtuin 1; TIM-3, T-cell immunoglobulin and mucin-domain-containing protein 3; TILs, tumor-infiltrating lymphocytes; TLR4, Toll-like receptor 4; TME, tumor microenvironment; TOM20, translocase of outer mitochondrial membrane 20; TOM22, translocase of outer mitochondrial membrane 22; TMRE, tetramethylrhodamine ethyl ester; USP14, ubiquitin-specific protease 14; VEGF, vascular endothelial growth factor.

This mechanism may reduce metabolic competition within the TME, thereby indirectly improving T-cell function. Importantly, OXPHOS inhibition may also decrease hypoxia and modulate signaling pathways that regulate immune checkpoint expression (84–86). For example, VLX600 impairs complexes I, II and IV of the electron transport chain, inducing profound mitochondrial dysfunction in preclinical CRC models (99) whereas ASP4132 has entered phase I clinical evaluation in patients with advanced solid tumors, including CRC, demonstrating acceptable tolerability at lower dose levels (100). In addition, inhibition of the SIRT1/PGC-1α axis has emerged as another strategy to suppress mitochondrial biogenesis and OXPHOS in patient-derived colonospheres (102).

Another promising approach consists of targeting metabolic pathways associated with lactate production and amino acid metabolism, which contribute to immune suppression within the TME. Inhibition of LDH-A or treatment with the LDH inhibitor oxamate synergizes with phenformin to promote apoptosis and suppress tumor growth in CRC models (105), while LDH-A silencing has been reported to enhance the efficacy of anti-PD-1 immunotherapy (106). Likewise, targeting hypoxia signaling through HIF-1/2 inhibition increases CD8^+^ T-cell and NK-cell infiltration and potentiates anti-CTLA-4 therapy in preclinical CRC models (107). Similarly, modulation of glutamine and arginine metabolism represents another promising strategy. Inhibition of ARG2 restores antitumor immunity in combination with immune checkpoint blockade in experimental CRC (108), whereas clinical evaluation of the dual ARG1/ARG2 inhibitor OATD-02 and the glutamine antagonist DRP-104 in combination with anti-PD-1/PD-L1 therapy is currently underway in patients with advanced solid tumors, including CRC (NCT05759923, NCT04471415).

Another approach may consist in targeting pathways involved in mitochondrial quality control, such as mitophagy and mitochondrial biogenesis, which may disrupt CRC cell adaptive capacity. Indeed, the inhibition of mitophagy can promote the accumulation of damaged mitochondria, increase oxidative stress and promote apoptotic cell death of CRC cells. Several molecules have been proposed to interfere with this process, including piR-823 and USP14 inhibition and strigolactone analogues that disrupt autophagosome-lysosome fusion, all of which impair mitochondrial quality control and suppress CRC progression in preclinical models (110–112).

Moreover, TME may also be targeted to reduce tumor-driven nutrient depletion, as well as hypoxia, by impairing HIF signaling and pro-tumorigenic metabolite accumulation (e.g., lactate). Targeting intercellular mitochondrial transfer may also represent a potential therapeutic strategy to limit CRC cell metabolic adaptation (49, 94). In particular, inhibiting mechanisms such as tunneling nanotube formation or mitochondrial trafficking could prevent the restoration of mitochondrial function, increase the sensitivity to metabolic stress and synergize with therapies targeting intrinsic mitochondrial pathways or immunotherapy (84). For example, inhibition of oxidized HMGB1-dependent mitochondrial transfer from mesenchymal stromal cells to CRC cells reduces tumor stemness in preclinical models, further supporting mitochondrial trafficking as a therapeutic target (84).

However, it would be important to employ selective targeting strategies toward CRC cells, while sparing normal colonic epithelial cells and immune cells, due to the systemic nature of mitochondrial metabolism. Indeed, immune cells require intact mitochondrial function to promote anti-tumor response, in particular CD8^+^ T-cells. This can be achieved by promoting mitochondrial biogenesis through activation of regulators, such as PGC-1α, as well as reducing oxidative stress to preserve mitochondrial integrity or supporting metabolic flexibility to enable sustained effector function (113). However, these strategies remain largely at the preclinical stage, and their efficacy and safety in CRC patients have yet to be established.

Notably, mitochondrial-centered restorative strategies are also being investigated, primarily in preclinical settings to counteract exhaustion and limited persistence in CAR-T-cell therapy, highlighting the potential of targeting mitochondrial metabolism to sustain immune cell fitness (114).

The future clinical translation of these strategies will require robust biomarkers capable of capturing the complexity of this conceptual mitochondrial checkpoint and predicting responses to immunotherapy (**Table 3**). Tumor-intrinsic mitochondrial signatures (e.g., OXPHOS-related genes, mtDNA copy number and mutational burden), as well as activation of mitochondrial stress and unfolded protein response pathways, may provide insights into tumor metabolic states, although their predictive value should be integrated with immune-related parameters (115, 116). In addition, the assessment of immune cell mitochondrial fitness could also be used as a predictive marker of immunotherapy response. Of note, advanced technologies such as single-cell transcriptomics and metabolic imaging may enable the evaluation of these features within the TME, while non-invasive biomarkers, including circulating cell-free mtDNA, systemic metabolic profiles and oxidative stress markers (117, 118), may provide dynamic information on tumor metabolism and immune status during treatment.

**Table 3:** Potential mitochondrial biomarkers predicting responses to therapy.

Biomarker	Category	Sample	Method of measurement	Proposed clinical use	CRC validation
OXPHOS-related molecular signature	Tumor	Tissue	scRNA-seq	Potential predictive	Preclinical
mtDNA copy number	Tumor	Tissue	qPCR	Prognostic; potential predictive	Clinical
mtDNA mutational burden	Tumor	Tissue	WES	Potential predictive	Clinical
Mitochondrial stress	Tumor	Tissue	scRNA-seq	Potential predictive and prognostic	Clinical
Immune-cell mitochondrial fitness	Immune	Tissue	Flow cytometry, Seahorse assay	Potential predictive	Not validated
Cell-free mtDNA	Serum	Circulating	qPCR/NGS	Prognostic	Clinical
Systemic metabolic profile	Plasma/serum	Circulating	Metabolomics	Potential predictive	Not validated
Oxidative stress markers	Serum	Circulating	d-ROMs test	Pre-diagnostic and prognostic	Clinical

CRC, colorectal cancer; d-ROMs, derivatives of reactive oxygen metabolites; mtDNA, mitochondrial DNA; NGS, next-generation sequencing; OXPHOS, oxidative phosphorylation; qPCR, quantitative polymerase chain reaction; scRNA-seq, single-cell RNA sequencing; WES, whole-exome sequencing.

Given the complexity and heterogeneity of mitochondrial regulation across tumors, immune compartments and microenvironmental contexts, single biomarkers are unlikely to fully capture the functional status of the proposed mitochondrial checkpoint. Consequently, integrated models combining tumor metabolic signatures, immune cell mitochondrial fitness, TME characteristics, such as hypoxia and metabolite accumulation, together with established biomarkers, such as MSI status and tumor mutational burden (TMB) may offer greater predictive power for patient stratification and therapeutic decision-making.

## 6 Conclusion and future perspectives

Accumulating evidence suggests that mitochondrial dysfunction represents an important regulator of tumor-immune interactions in CRC due to its ability to influence metabolic adaptation, immune cell function and responsiveness to immunotherapy. Such alterations affect cancer, immune and stromal cells within the TME, supporting the concept of a mitochondrial checkpoint that contributes to immune evasion and therapeutic resistance. In particular, mitochondrial plasticity enables CRC cells to adapt to environmental and therapeutic stress through metabolic rewiring, mitophagy, mitochondrial biogenesis, and intercellular mitochondrial transfer. Importantly, most of these mechanisms have not yet been systematically compared between dMMR/MSI-H and pMMR/MSS CRC, and clarifying whether they contribute differentially to the divergent immunotherapy responses represents a key priority for future research.

Despite growing interest in overcoming resistance to different therapeutic approaches in CRC, including immunotherapy, several important limitations remain. For example, much of the current evidence derives from preclinical studies or from tumor types other than CRC, whereas mechanistic studies addressing the impact of mitochondrial regulation on anti-tumor immunity in CRC are still limited. Moreover, mitochondrial dysfunction can exert both pro- and anti-tumor effects depending on the cellular context, duration of stress and tumor subtype, highlighting the complexity of targeting mitochondrial pathways *in vivo*. Similarly, although mitochondrial biomarkers could be strategic to predict the response to immunotherapy, their clinical use should consider tumor heterogeneity, metabolic states, and the lack of standardized approaches for their assessment. Therefore, while targeting mitochondrial metabolism represents an attractive strategy to improve immunotherapy efficacy in CRC, a more comprehensive understanding of its context-dependent nature is needed before these approaches can be translated into clinical practice. Future studies integrating spatial, metabolic and single-cell technologies, together with functional validation in clinically relevant CRC models and prospective clinical investigation, will be essential to determine whether targeting our proposed mitochondrial checkpoint framework can be translated into safe and effective therapeutic strategies for patients with CRC.
